# Augmentation of Antidiabetic Activity of Glibenclamide Microspheres Using S-Protected Okra Powered by QbD: Scintigraphy and In Vivo Studies

**DOI:** 10.3390/ph15040491

**Published:** 2022-04-18

**Authors:** Waleed Y. Rizg, N. Raghavendra Naveen, Mallesh Kurakula, Awaji Y. Safhi, Samar S. Murshid, Rayan Y. Mushtaq, Walaa A. Abualsunun, Majed Alharbi, Rana B. Bakhaidar, Alshaimaa M. Almehmady, Ahmad Salawi, Adel Al Fatease, Khaled M. Hosny

**Affiliations:** 1 Department of Pharmaceutics, Faculty of Pharmacy, King Abdulaziz University, Jeddah 21589, Saudi Arabia; wrizq@kau.edu.sa (W.Y.R.); wabuassonon@kau.edu.sa (W.A.A.); rbakhaidar@kau.edu.sa (R.B.B.); amnalmehmady@kau.edu.sa (A.M.A.); kmhomar@kau.edu.sa (K.M.H.); 2 Center of Excellence for Drug Research and Pharmaceutical Industries, King Abdulaziz University, Jeddah 21589, Saudi Arabia; 3 Department of Pharmaceutics, Sri Adichunchanagiri College of Pharmacy, Adichunchanagiri University, B.G. Nagar, Karnataka 571448, India; raghavendra.naveen@gmail.com; 4 Product Development Department, CURE Pharmaceutical, Oxnard, CA 93033, USA; 5 Department of Pharmaceutics, Faculty of Pharmacy, Jazan University, Jazan 82817, Saudi Arabia; asafhi@jazanu.edu.sa (A.Y.S.); asalawi@jazanu.edu.sa (A.S.); 6 Department of Natural Products and Alternative Medicine, Faculty of Pharmacy, King Abdulaziz University, Jeddah 21589, Saudi Arabia; samurshid@kau.edu.sa; 7 Department of Pharmaceutics, College of Clinical Pharmacy, Imam Abdulrahman Bin Faisal University, Dammam 31441, Saudi Arabia; rymushtaq@iau.edu.sa; 8 Department of Pharmaceutical Chemistry, Faculty of Pharmacy, King Abdulaziz University, Jeddah 21589, Saudi Arabia; maaalharbi1@kau.edu.sa; 9 Department of Pharmaceutics, College of Pharmacy, King Khalid University, Abha 62529, Saudi Arabia; afatease@kku.edu.sa

**Keywords:** sustainability of natural resources, microsphere, thiolation, mucoadhesion, glibenclamide, central composite design, okra

## Abstract

Successful drug delivery by mucoadhesive systems depends on the polymer type, which usually gets adherent on hydration. The intended polymers must sustain the association with biomembranes and preserve or accommodate the drug for an extended time. The majority of hydrophilic polymers tend to make weak interactions like noncovalent bonds, which hampers the positioning of dosage forms at the required target sites, leading to inefficient therapeutic outcomes. It is possible to overcome this by functionalizing the natural polymers with thiol moiety. Further, considering that S-protected thiomers can benefit by improving thiol stability at a broad range of pH and enhancing the residence period at the required target, 2-mercapto-nicotinic acid (MA) was utilized in this present study to shield the free thiol groups on thiolated okra (TO). S-protected TO (STO) was synthesized and characterized for various parameters. Glibenclamide-loaded microspheres were formulated using STO (G-STO-M), and the process was optimized. The optimized formulation has shown complete and controlled release of the loaded drug at the end of the dissolution study. Cell viability assay indicated that the thiolated S-protected polymers gelated very well, and the formulated microspheres were safe. Further, G-STO-M showed considerable in vivo mucoadhesion strength. The glucose tolerance test confirmed the efficacy of STO formulation in minimizing the plasma glucose level. These results favor S-protection as an encouraging tool for improving the absorption of poorly aqueous soluble drugs like glibenclamide.

## 1. Introduction

The concept of mucoadhesion and mucoadhesion polymers originated in the 1980s. It is an intriguing approach for delivering drugs at specified target sites or the absorption window. Upon hydration they gelate, the mucoadhesive polymers get adherent with the mucus membrane and are found to have persistent contact and prolonged residence time [1]. Despite some exceptions, mucoadhesive gastric systems have not attained their full potential. The favorable outcome of much-earlier-generation mucoadhesive polymers when gelated was constrained by their inadequate adherence to the gastrointestinal tract by weak hydrogen bonds or ionic and Van der Waals forces. Thus, they were unable to promise the emplacement of dosage forms [2,3]. Thiolated polymers are one of the new viable mucoadhesive polymers [4] that are a favorite type of polymer excipients. Thiomers, in contrast to entrenched polymers, are more competent and can form strong molecular bonds through thiol/disulfide exchange reactions with membrane subdomains that are rich in cysteine when they gelate on hydration [5,6]. Based on the requirement, thiomers localize the dosage forms for an increased time with good biodegradation.

The oxidation state of the thiol moiety can modify the interactivity with mucus glycopeptides (cysteine-rich subdomains) and hence affects the mucoadhesive efficacy [7,8]. Thiolated polymers are S-protected by the analogs of mercaptopyridine like 2-mercapto-nicotinic acid (MA), which enhances the thiol group’s reactivity by shrinkage affecting the electrons in the pyridine π framework when they gelate [9].

The use of natural gums and mucilage of plants turns domineering into excipients for pharmaceuticals, particularly when formulating a controlled release drug system [10]. These substances’ physical and chemical characteristics can readily be modified to fulfill the criteria of an ideal drug delivery method [11]. Abelmoschus esculentus (L.) Moench (commonly called okra) of the Malvaceae family is primarily cultivated in tropical and subtropical regions [12]. Okra gum is employed in the preparation of mucoadhesive beads [13], buccal films [14], and other controlled matrix delivery systems [15]. Okra gum has an excellent mucoadhesion feature when hydrated, but still, for employing in mucoadhesion delivery systems, it is necessary to improve the adhesion potential by appropriate methods like thiolation [16]. However, no research has demonstrated the synthesis of S-protected thiolated okra gum (STO) that gelates on hydration and evaluated its usage in the formulation of mucoadhesive drug delivery systems.

Glibenclamide is an oral antidiabetic agent that belongs to the class sulphonylurea. It stimulates the release of insulin from pancreatic beta cells and decreases glucose levels in plasma. It can also raise insulin levels by decreasing its clearance in the liver [17]. As per the biopharmaceutics classification system, glibenclamide is a class II drug having less solvability and greater penetrability [18]. It is not soluble in an aqueous medium, and hence the dissolution is regarded as the rate-limiting step for absorption. It is a weak acid drug with 5.3 pKa, so its solubility depends on the pH, and absorption is presumed to occur well in the upper GI tract [19]. The drug’s acidic behavior and pKa value indicate that the significant absorption site is the upper GI tract. This renders a narrow absorption window that could describe variability and less drug bioavailability after intake of conventional oral tablets. In due course, it is beneficial to develop a floating system that assures continuous drug release in the stomach and regulates the drug release with the potential improvement of the oral bioavailability of the drug. The current work seeks to synthesize and apply STO to formulate mucoadhesive microspheres loaded with glibenclamide that gelate overhydration and owe strong mucoadhesion properties to biomembranes and aid for enhanced targeting, bioavailability, and efficacy.

## 2. Results and Discussion

Okra gum was thiolated successfully by forming an ester bond between okra gum (-OH group) and thioglycolic acid (-COO groups) with a yield of around 75%. The end product was creamy to brown and had better flow properties. FTIR spectrum of TO consisted of all the peaks similar to those of okra gum and exhibited a sharp peak at a frequency of 2600 cm^−1^, denoting the existence of a thiol moiety.

### 2.1. Synthesis and Characterization of STO

S-protected thiomers render the benefit of improved thiol stability over a broad range of pH and also permit their further usage with microspheres generating technique in the existence of oxygen. Hence, the STO was developed to enhance stability and mucoadhesion features forming gelation around while hydrated. Since the TO can be oxidized easily in the solution form or the existence of oxygen, it is restrained in the formulation of multiarticulate systems. TO was S-protected by combining with MA, the free thiol moiety, and preventing their oxidation. A disulfide-thiol interchange reaction obtained S-protection of thiol groups betwixt TO and MA [20]. Table 1 demonstrates the physicochemical characterization of okra gum and STO. A significant change was not observed concerning these properties, except for the formation of a thick gel with water for STO.

**Figure 1:**
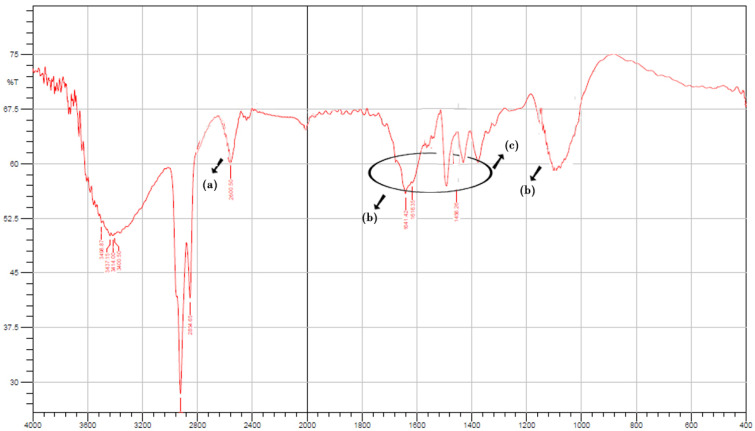
FTIR spectrum of STO (**a**) thiol functionalization of OG, (**b**,**c**) S-protected thiomers exhibiting wide peaks between 1650 and 1450 cm^−1^ due to –C=C stretch vibrations (**d**) C-H wagging between thiomer and aromatic ligand.

FTIR studies were performed to validate the integration between thiomers and aromatic ligands. Figure 1 shows the FTIR spectrum of TO and S-protected okra gum. A specific band near 3030 cm^−1^ was associated with the stretching vibration of an aromatic (-C-H) group, which was observable in the IR spectrum of the STO (Figure 1a). At 1166 cm^−1^, C-H wagging was observed (Figure 1d). Additionally, the S-protected thiomers exhibited wide peaks between 1650 and 1450 cm^−1^ due to –C=C stretch vibrations, generally four bands of varied intensity, which also indicated the existence of the aromatic structures (Figure 1b,c).

**Figure 2:**
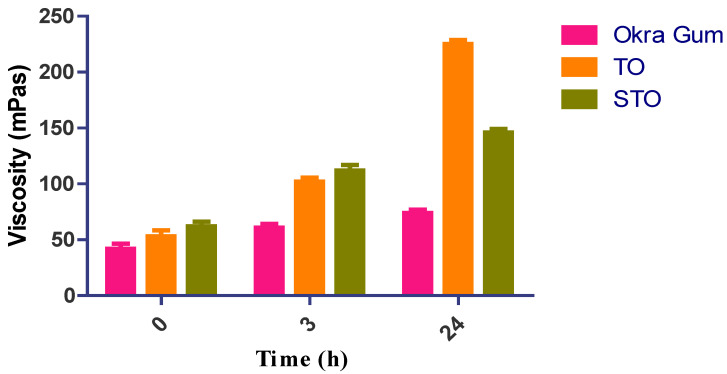
Rheological studies of okra gum, TO, and STO.

The rheological examination was conducted on a combined plate-plate rheometer to evaluate the viscoelasticity of every polymer sample and, accordingly, an interpretation of their cytotoxicity could also be correlated. As a result of adding thioglycolic acid to okra gum, the thiomers’ dynamic viscosity improved by at least 1.67-fold (TO); 2.66-fold (STO) in 3 h. Surprisingly, a 5.35-fold increase in the viscosity was noted for TO after 24 h of study, and no significant changes were identified in the viscosity of okra gum and the S-protected thiomers. At physiological pH following a definite time, thiolated polymers can undergo oxidation and may form intramolecular disulfide bonds. Due to this cross-linking, their viscoelastic properties get altered concerning the function of time. Additionally, it may be noted that the higher the number of immobilized thiol moieties on the polymer, the more significant the improvement in viscosity, which was found to be similar to the results of Sakloetsakun et al. [21]. However, in STO, because of the fewer free and connected thiol groups, this disulfide bond cannot be formed to a similar degree as their corresponding thiomers. Kafedjiiski et al. demonstrated that thiol groups could rapidly form disulfide bonds due to proximity compared to isolated residual thiol groups [22]. This study’s results with regard to apparent viscosity are shown in Figure 2.

Ellman’s assay was conducted to determine the number of free thiols and oxidized thiol groups present on the structural framework of thiolated and S-protected okra gum. The test results are noted in Table 2. The disulfide bond in Ellman’s reagent can interact with free thiols on the polymer backbone, leading to a yellow product, 2-nitro-5-thiobenzoic acid, which can be measured easily at 450 nm. Using NaBH_4_, the total number of –SH was determined to reduce all the disulfides quantified with Ellman’s reagent. The number of groups of oxidized thiols were calculated by subtracting the free thiol groups from the total amount of thiol groups. TO exhibited mean thiol group content of 128.31 ± 4 μmol/g and disulfide groups of 102.84 ± 5 μmol/g. The determination of MA conjugated in STO was performed by adding glutathione, which releases MA by disulfide-thiol exchange reaction and is eventually measured by photometry (Table 2). The conjugate revealed MA content of 119.63 ± 7 μmol/g, indicating that around 90% of all thiol groups were S-protected. Hence, S-protection of TO could maintain the stability of the carrier against oxidation before reaching the lining of mucus.

### 2.2. Formulation of STO-Loaded Glibenclamide Microspheres

The central composite design of response surface methodology (RSM) was used to evaluate the optimum degree of the factors chosen and their interconnection in achieving desired EE and mucoadhesion properties due to gelation on hydration. Fifteen experimental runs were conducted, and the responses are given in Table 3. For all the trail preparations, EE was noted between 47 and 87, while mucoadhesion strength was observed in the range of 21 and 82. The results obtained were scrutinized for individual responses, and statistical modeling, i.e., ANOVA and fx were applied to find the effect of parameters.

For all the responses, a quadratic model was opted, depending on the sum of squares (Type I) and fit summary (adjusted and predicted R^2^) (Table 4). A quadratic model was chosen (high order polynomial), where the auxiliary terms are noticeable, and the model is not aliased. The predicted R^2^ for both the responses of 0.9176 and 0.9241 was in agreement with the adjusted R^2^ of 0.9738 and 0.9797, correspondingly, as the disparity was less than 0.2. Adequate precision measures the S/N ratio. Usually, a fraction above four is desirable. Lag time and T-95% CDR show this ratio of 30.2872 and 35.6278, indicating a relevant signal, therefore affirming the model’s efficiency to operate the design space. The Model F-value of both the responses was observed to be 90.29 and 116.97, denoting that the model was appreciable. Only a probability of 0.01% existed to show that the high F-value occurred because of noise.

The coefficient of variation (CV) value ensures the model’s repeatability. The reproducibility of the present model was found to be CV < 10 %. Relatively low CV values were observed in the study, which assures the accuracy and reliability of the model. Insufficient fit can lead to an ineffectual model to depict the entire data (Table 5). Thus, lack of fit is essential to determine that the model-generated equations predict the outcomes rationally. All the *p* values of EE and mucoadhesion were negligible, so the selected model was pertinent for the study [23].

ANOVA was performed to test the inference of quantitative effects of the fact factors. Polynomial equations were obtained by processing the data through multiple regressions. ANOVA results outranged the statistical significance developed by the quadratic equation; further, the *p*-value was <0.0500, denoting the importance of model terms. The test design specified that lag time was majorly affected by (a) the antagonist effect of polynomial terms of B with a *p*-value of 0.0004 and (b) the synergistic effect of A and B, with *p*-values of <0.0001 and 0.0002, correspondingly, with inflated A effects. Response 2 was profoundly contrived by (i) the antagonist effect of polynomial term of A with *p*-value of 0.0013 and (ii) the synergism effect of A and B with *p*-values of <0.0001 and 0.0026, correspondingly, and amongst the important variables, term A affected the mucoadhesion with high enormity. ANOVA co-efficients with their *p*-values for both the responses were displayed in Table 6. The equations developed from the responses for the best feasible model are noted below:

EE = +68.00 + 12.81 A + 4.88 B + 1.25 AB + 0.3125 A^2^ − 4.69 B^2^ In vitro mucoadhesion = +70.40 + 18.08 A + 3.94 B + 1.00 AB − 10.20 A^2^ + 0.3000 B^2^

**Figure 3:**
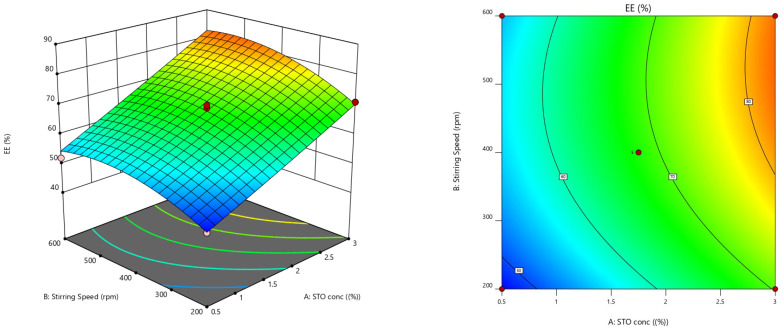

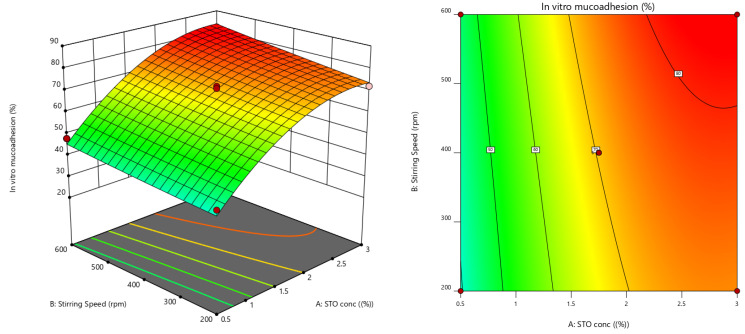
Response surface graphs for EE and in vitro mucoadhesion (3-dimensional and contour).

**Figure 4:**
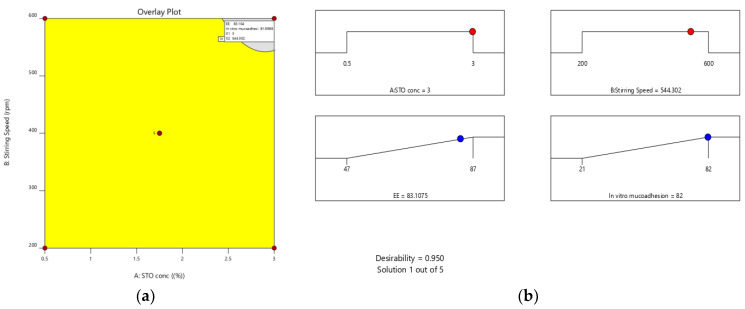
(**a**) Overlay plot and (**b**) desirability bar graph for an optimized result.

Furthermore, the effect of individual modifiers on responses was scrutinized and illustrated by RSM [24]. Figure 3 contains the response’s relation with the variables, a contour plot that ensures the variable effects. RSM was used to determine and explain the effect of the nondependent variables on the obtained individual responses. Three-dimensional response surface graphs are critical to demonstrate the interaction and the main effect. The acquired responses are visualized by employing contour plots [25]. The global desirability (D) function optimized the model order. Every response was laid a limit to the maximum to obtain an inlay graph to augment the nondependent variables. All three possible independent variables were encompassed in the design for optimization. The independent variables (optimal level) represented a maximum of 0.950 desirabilities (Figure 4a) value for both responses in the desirability function plot (Figure 4b). Hence, implementation of this setting aids in obtaining the EE of 83.11% and in vitro mucoadhesion of 82%. By using these optimized concentrations a G-STO-M formulation was formulated. For comparative purposes, a similar formulation (G-TO-M) was prepared using TO instead of STO.

### 2.3. Characterization and Evaluation of G-STO-M

**Figure 5:**
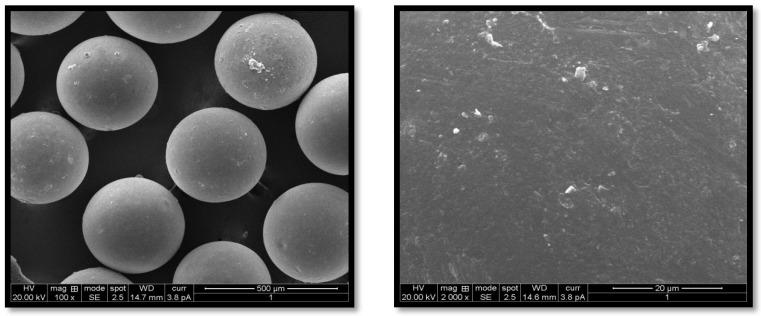
SEM of the optimized formulation at various magnifications.

G-STO-M was formulated using optimized conditions and evaluated for EE, and in vitro mucoadhesion and the results were found to be 82.54 and 83.64%, respectively. High STO concentration contributed to maximizing the EE, as the polymer got precipitated on the dispersed phase; this would not permit the drug to cross the surface border and raise the viscosity, which further reduces the diffusion of the drug inside the polymer droplets. Concerning stirring rate, an increase in stirring speed dissects the emulsion into smaller droplets, leading to the formation of smaller microspheres. These small droplets permit drug diffusion before it becomes hard and forms a microsphere, which minimizes the EE of the system. The stirring rate had a lesser effect on mucoadhesion than the concentration of STO, which may be ascribed to a smaller particle size at more incredible speeds, leading to a higher surface area. In contrast, the internal-to-external phase ratio did not have any effect. The surface morphology of G-STO-M with a presence of STO was noted to be discrete, globular, free-flowing, having a rough surface (Figure 5). The rough surfaces may result from microsphere accumulation and quick gelation of a thiomer.

### 2.4. Drug Release Studies

**Figure 6:**
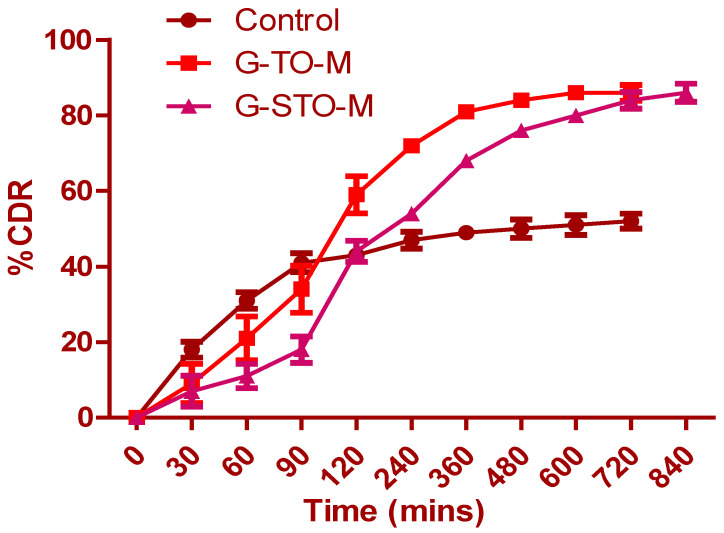
In vitro dissolution study for control (pure glibenclamide), G-TO-M, and G-STO-M.

Figure 6 shows that drug release from pure glibenclamide (control), G-TO-M, and G-STO-M was highly dependent on TO and STO concentrations. Drug release from the control formulation was incomplete, and it was observed that only 47.23% of glibenclamide was released at the end of 240 min. Subsequently, there was no significant change in the release. A similar drug release pattern was observed with TO and STO microsphere formulations. However, the formulation contained an S-protected polymer that showed more controlled and complete drug release by the end of 840 min due to the formation of a hydrogel network upon hydration. STO concentration in the microspheres led to increased thickening of the coat enclosing the drug particles, thus increasing the distance traveled in drug release. The increased polymer quantity by forming a hydrogel network can result in low porosity of the polymeric phase and impedes burst release.

### 2.5. Cell Viability Analysis by Resazurin Assay

**Figure 7:**
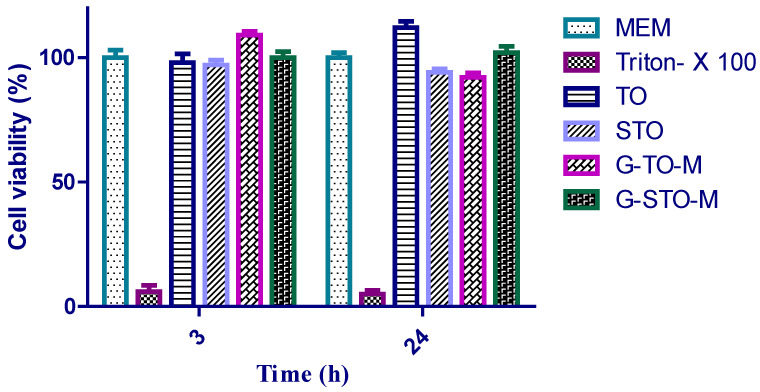
Cell viability studies.

Cell viability analysis of TO, STO, G-TO-M, and G-STO-M was conducted on the Caco-2 cell cultures using a Resazurin assay to determine cytotoxicity. Resazurin is a weakly fluorescent blue dye that indicates cell viability; it can be reduced irreversibly by living cells to resorufin, which is characterized by pink color and high fluorescence. It is primarily employed for in vitro cytotoxicity studies to determine cellular metabolic activity. Resorufin can easily be identified by fluorimetry with 540 and 590 nm emission wavelengths. Cells were given with the above samples for 3 and 24 h. The results were depicted as histograms in Figure 7, demonstrating that all the thiolated S-protected polymers and their formulations were not cytotoxic as the cell viability was very high. Additionally, no remarkable differences were noted after 3 and 24 h. Following 24 h of incubation, statistically significant differences regarding cell viability were noted for the microsphere referred to 3 h of incubation. Presuming that a polymer exhibits a higher number of covalently bound thiol groups, closer thiol moieties on the same chain can react rapidly among themselves rather than with thiols on other polymer chains. This could be owed to the higher viscoelastic characters of thiomers and their subsequent reduction in cell viability. Hence, the newly prepared S-protected polymer could be viewed as safe for in vivo usage.

### 2.6. In Vivo Mucoadhesion Studies

**Figure 8:**
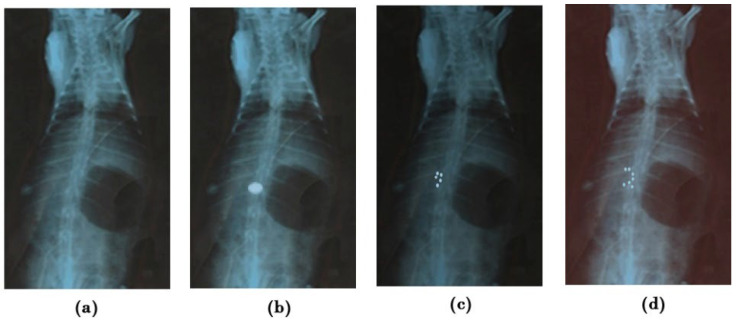
X-ray radiograms (**a**) before administering (**b**–**d**), showing mucoadhesion of G-STO-M at 1, 4, and 8 h, correspondingly.

The X-ray method studied the mucoadherent behavior of G-STO-M in the rat stomach. Before administering a formulation, an X-ray photograph was taken (Figure 8a). Following 1 h post-administration, the next X-ray photographs were taken (Figure 8b). All the microspheres (500 mg)were found to accumulate in one region of the stomach and hence look like the impression of the tablet. The radiographic pictures attained at the end of 4 h disclosed that the group of microspheres was lightly changed in their position and segregated into five different groups, but the position was restricted to the mucosal region of the stomach. As the time extended, more groups of the formulation were evident. The formulation was capable of resisting peristaltic movements of the GIT even at the end of 8 h, as it established a secured disulfide bridge with the mucosa (Figure 8c,d).

### 2.7. Glucose Tolerance Test

**Figure 9:**
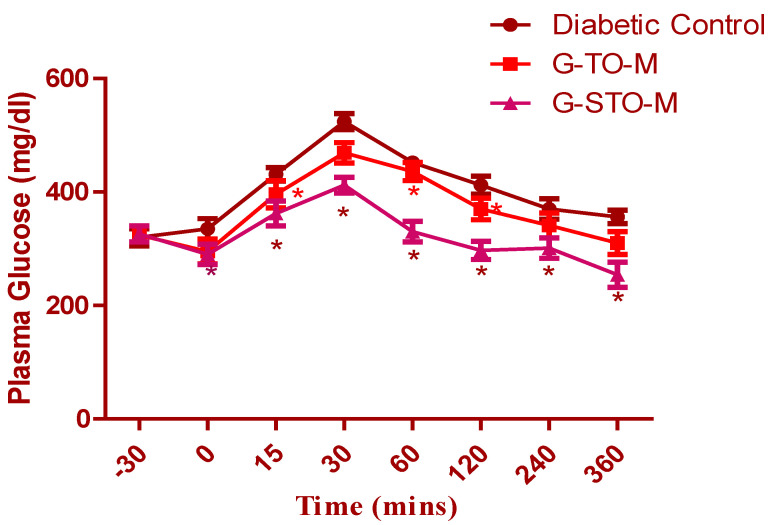
Results of oral glucose tolerance test (* *p* < 0.05).

The glucose tolerance test was conducted on diabetic (alloxan-induced) rats (Figure 9). The data is represented as mean ± SEM (*n* = 6). Considerable deviations were noted in the groups upon statistical validation with one-factor ANOVA and subsequent Dunnett’s test for multiple comparisons (* *p* < 0.05). The G-STO-M formulation exhibited excellent control of plasma sugar levels due to better penetration of glibenclamide, as a consequence of improved permeability characters by the S-protected thiomer.

## 3. Materials and Methods

### 3.1. Materials

Aurobindo Pharma, Hyderabad, India, generously presented glibenclamide. Sodium trimetaphosphate, Span 80, and Tween 80 were procured from Loba Chemie Pvt Ltd.; Mumbai, India. 2-mercapto nicotinic acid (MA) and Triton X-100 were acquired from SD Fine-Chem Ltd. (Mumbai, India). The other chemicals and solvents used were of analytical grade.

### 3.2. Synthesis and Characterization of TO

Okra gum was isolated and thiolated by esterification using thioglycolic acid, as previously demonstrated by our research team [26]. Synthesized TO was characterized and evaluated for mucoadhesion strength of gel upon hydration.

### 3.3. Synthesis and Characterization of S Protected Thiolated Okra (STO)

To prepare STO, Thiolated okra was combined with aromatic MA (dimeric form) through a thiol-disulfide exchange reaction [27]. In brief, 400 mg of TO was dissolved in 15 mL of water, and further, dimeric MA of 100 mg was mixed with the solution by constant stirring, and a pH of 8.0 was maintained by adding 1 M sodium hydroxide. The obtained mixture was agitated for 4 h at ambient conditions away from light. The product was subjected to dialysis through a cellulose dialysis tube (MWCO 100–500 Da, Spectra/Por). The resultant solution was allowed for freeze-drying to achieve the protected product (STO). The final STO was preserved at 4 °C until further use.

### 3.4. Characterization of S-Protected Thiolated Okra

Okra gum and STO were assessed for different physicochemical properties like solubility, pH, and moisture content, and tested for foreign matter and arsenic.

#### 3.4.1. Fourier Transform Infrared Spectroscopy (FT-IR)

The FTIR spectral readings were recorded at room temperature with an IR spectrophotometer (Perkin Elmer Instruments, North Billerica, MA, USA). This was performed to evaluate the peak patterns and for comparative use [28]. The spectral readings of TO and STO were recorded at a wavelength of 400–4000 cm^−1^ with a KBr disc.

#### 3.4.2. Rheological Investigations

All compound’s viscoelastic characters were determined using a plate rheometer (RotoVisco RT20, Haake GmbH, Karlsruhe, Germany). Briefly, 1 mL of deionized water was used to hydrate the sample of each 10 mg polymer, followed by dilution with phosphate buffer (pH 7.4) to achieve a solution of 0.5% (*m*/*v*) concentration. After adding a buffer solution and an equilibration time of 3 h or one day at 37 °C in an incubator, a volume of 500 μL of every polymer solution was placed in the plate viscometer. For all the samples, apparent viscosity (η) was determined. The two plates were kept apart at 0.5 mm, the tangential stress was kept between 0.5 and 500 Pa, and the investigation was done at 37 °C temperature.

#### 3.4.3. Quantitative Analysis of Thiol/Disulfide/MNA Groups

The number of thiol and disulfide groups of STO was quantitatively estimated with the formerly reported procedures (partial and total Ellman’s assay) [29]. To estimate MA linked to a polymer, a sample of 0.5 mg was allowed to dissolve in 500 μL phosphate buffer (0.5 M) at pH 8. Freshly prepared reduced glutathione solution (10 mg/5 mL) was used to release MA for its quantification, and the resultant mix was incubated at 37 °C for 90–120 min. The absorptivity of free MA, generated by a thiol-disulfide interchange reaction with glutathione, was determined by a Perkin Elmer VICTOR X3 Multilabel Plate Reader at 354 nm. To determine the absorptivity of unbounded MA, a control was used, excluding the glutathione solution. TO was used for comparing purposes by the procedure mentioned above.

### 3.5. Formulation of Microspheres

The measured quantity of STO was dissolved in 50 mL of 1 M fresh sodium hydroxide solution. Using a mechanical stirrer, agitation was done for 15 min to form a homogenous mixture. An adequate quantity of glibenclamide was dispersed into the resultant solution. A further required quantity of sodium trimetaphosphate was added and continuously stirred for 15 min. The formed dispersion was expelled drop by drop with a syringe into preheated corn oil and positioned on a magnetic stirrer. To prevent the accumulation of droplets, a sufficient quantity of surfactants like Span 80 and Tween 80 was added to corn oil. Thus, the obtained microspheres were segregated by centrifugation. Further, they were filtered and cleaned with propanone to drain the additional corn oil. The segregated microspheres were subjected to air drying for 48 h.

#### Optimization Using Response Surface Methodology and Various Statistical Applications

The process parameters chosen were STO (X_1_) concertation and stirring speed (X_2_) at five stages coded as −1.414, −1, 0, +1, and +1.414. These variables were standardized for entrapment efficacy (EE) (Y_1_) and in vitro mucoadhesion nature (Y_2_). Design Expert V.12 was used to implement the central composite model, providing 15 experimental trials. Table 7 depicts the entire work plan interns of coded and real values of selected variables and restrains of dependent factors [30]. Quadratic regression was applied to measure the response in every trial, and an investigation was performed.

### 3.6. Evaluation of Trial Batches

#### 3.6.1. EE

The formulated microspheres (50 mg) were broken in a glass mortar, and the powder was dispersed in 50 mL of phosphate buffer (pH 7.4). The obtained solution was subjected to sonication for around half an hour in a bath sonicator and left overnight [31,32]. Following 24 h, filtration was done, and a spectrophotometry study of the filtrate was performed at 231 nm. The entrapment efficiency can be determined using the formula:

EE = practical drug content/theoretical drug content × 100

#### 3.6.2. In Vitro Mucoadhesion Test for Microspheres

The mucoadherant features of microspheres upon hydration were determined by the in vitro wash-off test described by Sativa et al. [33]. A 1 × 1 cm piece of rat gastric mucosa was fastened with a thread on a glass slide of 3 by 1 inches. Microspheres (500 mg) were dispersed on the damp, washed tissue specimen, and the developed slide was put up onto a ridge of the disintegrating test apparatus (USP) having 28–29 reciprocations per minute. The disintegrating test machinery was run so that the specimen was consistently moved perpendicularly in a jar of counterfeit gastric fluid USP with 1.2 pH maintained at 37 ± 0.5 °C. By the end of 30 min and at every one hour, the number of microspheres that were still adherent on the specimen tissue was noted until the completion of 10 h.

### 3.7. Preparation, Characterization, and Evaluation of Optimized Formulation

The optimized formulation (G-STO-M) was prepared using optimized concentrations of STO and stirring speed. One more formulation was prepared using TO using the same methodology as that of G-STO-M for comparative purposes.

#### 3.7.1. Microscopy (SEM) 

SEM was conducted to study the morphology features of the optimized microsphere formulation (Jeol JSM-6350, Tokyo, Japan) [34]. The samples for SEM were prepared by mounting the microsphere onto an aluminum stub before coating for 70 s. The stubs were then coated with gold-palladium under an argon atmosphere using a gold sputter module in a high-vacuum evaporator. The coated samples were then randomly scanned, and photomicrographs were taken.

#### 3.7.2. Drug Release Studies

An in vitro release test was conducted with a USP XXIV basket type apparatus containing 900 mL of simulated gastric fluid (pH 1.2) as dissolution medium at 37 ± 0.5 °C which was operated at 100 rpm [27]. A quantity of microspheres equal to 20 mg of glibenclamide was used for the study. The dissolution media sample of 5 mL was drawn at prefixed time gaps, passed through a membrane filter of 0.45 µm pore size, diluted as per requirement, and studied at 231 nm by spectrophotometry. After drawing the test samples, a fresh dissolution medium was displaced instantly.

### 3.8. Biological Studies—Cell Culture and Cell Viability

#### Cell Cultures and Viability Test by Resazurin Assay

The resazurin (Alamar blue) assay was conducted on Caco-2 cell cultures as reported earlier [20,35] with similar incubation conditions. Sample solutions of unaltered, thiolated, and protected okra gum were made individually, and their microparticles were developed using white MEM at a concentration of 1% m/v. White MEM and 1% m/v of Triton X-100 were correspondingly used as positive and negative controls.

### 3.9. In Vivo Studies

All the in vivo studies were conducted after obtaining approval from the animal ethical committee of the institution of the clinical laboratory center, Beni-suef, Egypt (Approval no. 18/3-02-22).

#### 3.9.1. In Vivo Mucoadhesion Study

The study was conducted with healthy albino rabbits of either sex bearing 2.5–3 kg of weight. The animals were subjected to 12 h of fasting and had access only to water throughout the study. A prior abdominal X-ray was done to confirm the absence of any radio-dense substances. Further, barium sulfate (15%) was integrated into the preparation (without the drug) to enable the microsphere’s visibility under X-ray. Around 500 mg of the formulation was ingested into the animal with a flexible tracheal tube together with 15 mL of water. Anesthesia was given to the animal, and abdominal X-ray scans were taken at distinct time intervals by positioning the animal in an upright posture with the assistance of a radiologist [36].

#### 3.9.2. Oral Glucose Tolerance Test

The institute’s animal ethics committee authorized the study protocol. Wistar albino rats of either sex with 150–250 g of weight were used for the study and subjected to 24 h of fasting (with water). Diabetes was induced by injecting mono-hydrate alloxan (120 mg/kg) into the peritoneum. The rats were provided with standard lab feed water and were monitored under optimized lab conditions. The animals were segregated randomly into three groups (n = 18). Group I: Positive control (nondiabetic rats); Group II (G-TO-M), and Group III (G-STO-M microspheres equal to 800 µg/kg of drug). A tolerance test was conducted for one week after alloxan administration (9.00–10.00 a.m.). A 50% Dextrose solution (2.5 g/kg body mass) was administered through the mouth using an orogastric tube to conscious rats. At a predetermined time, gaps of 0, 30, 60, and 120 min, blood was drawn from the rats’ lateral tail vein, and plasma glucose levels were estimated with a glucometer (Make: Contour TS).

### 3.10. Statistical Analysis

ANOVA was conducted using Bonferroni’s Test for multiple comparisons (version 5 Graph Pad Prism for Windows, Graph Pad Software, San Diego, CA) and *p <* 0.05.

## 4. Conclusions

Thiomers have the potential to form intermolecular and/or intramolecular disulfide bonds that can promote mucoadhesion strength, improve swelling upon hydration, and aid sustained drug release. Enhanced mucoadhesion potential was observed in the S-protection of thiolated polymers. STO was synthesized and characterized for rheological properties and the quantitative analysis of thiol/sulfide/MA groups. The preparation of glibenclamide microspheres was optimized using a central composite design. According to the desirability approach, a formulation consisting of 3% STO and 544 rpm can fulfill the requirements of optimum formulation for preparing G-STO-M. Optimized formulation was studied for drug release, cell viability, and oral glucose tolerance test. Cell viability studies confirmed that all the thiolated S-protected polymers and formulations were not cytotoxic, as cell viability was very high. The G-STO-M formulation showed better plasma glucose control, which could be credited to the presence of STO, which may have enhanced the penetration and absorbance of glibenclamide.

## Figures and Tables

**Table 1:** Comparison of physicochemical properties of okra gum and STO.

S. No	Property	Okra Gum	STO
1.	Appearance	Light brown color semi-granular powder	Dark brown color granular powder
2.	Solubility	In Water—Slightly soluble (forms light gel) Organic solvents—Insoluble	In Water—Slightly soluble (forms thick gel) Organic solvents—Insoluble
3.	pH (2% *w*/*v* solution)	5.8	6.4
4.	Moisture content	16.63%	15.89%
5.	Test for foreign matter	<0.1%	<0.1%
6.	Test for arsenic	<0.1 ppm	<0.1 ppm

**Table 2:** The quantity of thiol and disulfide groups was estimated by quantitative assays.

Sample	-SH	-S-S-	MNA
		(µmol/g)	
TO	128.31 ± 6.8	102.84 ± 12	–
STO	–	–	119.63 ± 14.5

**Table 3:** Projected trial batches and their responses for central composite design.

	Factor 1	Factor 2	Response 1	Response 2
Run	A: STO conc	B: Stirring Speed	EE	In vitro mucoadhesion
	(%)	rpm	%	%
2	−0.0177 (0) *	400	52	21
4	0.5	600	52	48
5	0.5	200	47	42
3	1.75	400	67	69
7	1.75	400	67	71
9	1.75	400	69	72
10	1.75	117.157 *	51	65
11	1.75	400	70	70
12	1.75	400	67	70
13	1.75	682.843 *	68	76
1	3	600	81	82
6	3	200	71	72
8	3.5177	400	87	78

* Stirring speed as suggested by Design Expert and Round off to 117 and 683. (* Factor concentration was set to zero as −0.0177 was practically not possible).

**Table 4:** Model summary statistics of selected responses.

	Source	Sequential *p*-Value	Lack of Fit *p*-Value	Adjusted R^2^	Predicted R^2^	
EE	Linear	<0.0001	0.0099	0.8647	0.7782	
	2FI	0.5946	0.0078	0.8546	0.7224	
	**Quadratic**	**0.0010**	**0.1595**	**0.9738**	**0.9176**	**Suggested**
	Cubic	0.2201	0.1550	0.9800	0.7613	Aliased
In vitro mucoadhesion	Linear	0.0006	0.0003	0.7318	0.5625	
	2FI	0.8351	0.0002	0.7035	0.5066	
	**Quadratic**	**<0.0001**	**0.0779**	**0.9797**	**0.9241**	**Suggested**
	Cubic	0.0124	0.2826	0.9951	0.9614	Aliased

**Table 5:** Model (quadratic) fit summary of the responses.

Parameter	PS	EE
Std. Dev.	1.92	2.44
Mean	65.31	64.31
C.V. %	2.94	3.80
Adeq. Precision	30.2872	35.6278
**Lack of Fit F-value**	2.98	9.36
**Lack of Fit** ***p*** **-value**	0.1595	0.0779
Model F-value	90.29	116.97
Model *p*-value	<0.0001	<0.0001

**Table 6:** ANOVA coefficients table for both the responses.

	Intercept	A	B	AB	A^2^	B^2^
**EE**	68	**12.8122**	**4.8802**	1.25	0.3125	**−4.6875**
***p*** **-values**		**<0.0001**	**0.0002**	0.2347	0.6810	**0.0004**
**In vitro mucoadhesion**	70.4	**18.0763**	**3.94454**	1	**−10.2**	0.3
***p*** **-values**		**<0.0001**	**0.0026**	0.4396	**<0.0001**	0.7553

**Table 7:** Total work plan interns of coded and real values of selected parameters and constraints of dependent factors for central composite design.

Selected Formulation Factors	Levels					Responses/Dependent Variables	Constraints
	−1.414	−1	0	+1	+1.414		
Concentration of STO (%)-X_1_	0.00	0.5	1.75	3	3.5177	EE (%)	Maximum
Stirring speed (rpm)-X_2_	117.157	200		600	682.843	In vitro mucoadhesion (%)	Maximum

## Data Availability

Data is contained within the article.
